# Complement Factor 3 Could Be an Independent Risk Factor for Mortality in Patients with HBV Related Acute-on-Chronic Liver Failure

**DOI:** 10.1155/2016/3524842

**Published:** 2016-04-06

**Authors:** Geng-lin Zhang, Ting Zhang, Yi-nong Ye, Jing Liu, Xiao-hong Zhang, Chan Xie, Liang Peng, Zhi-liang Gao

**Affiliations:** ^1^Department of Infectious Diseases, The Third Affiliated Hospital of Sun Yat-Sen University, Guangzhou 510630, China; ^2^Guangdong Provincial Key Laboratory of Liver Disease, The Third Affiliated Hospital of Sun Yat-Sen University, Guangzhou 510630, China; ^3^Department of Ultrasound, The Third Affiliated Hospital of Sun Yat-Sen University, Guangzhou 510630, China; ^4^Department of Infectious Diseases, Foshan Hospital of Sun Yat-Sen University, Foshan 528000, China; ^5^Key Laboratory of Tropical Disease Control, Sun Yat-Sen University, Ministry of Education, Guangzhou 510630, China

## Abstract

The complement is thought to be involved in the pathogenesis of multiple liver disorders. However, its role in patients with HBV related acute-on-chronic liver failure (HBV-ACLF) remains unclear. Serum levels of the third and fourth complement components (C3, C4) and complement function (CH50) were examined in this prospective, observational study. Associations between their expression and disease activity were analyzed. Survival was analyzed by Kaplan-Meier curves. Predictors of clinical outcome were determined by Cox regression analysis. C3, C4, and CH50 levels were significantly lower in HBV-ACLF patients compared to controls. C3, C4, and CH50 levels were negatively correlated with Tbil levels but positively associated with PTA levels. C3 levels were negatively associated with MELD-Na. C3 levels were significantly lower in HBV-ACLF patients who died compared to patients who survived. In a median hospital stay of 39 days, mortality occurred in 41 patients with a progressive increase based on C3 grade (*P* = 0.008). The actuarial probability of developing mortality was significantly higher in patients with low C3 grade compared to those with high C3 grade (*P* < 0.001). Multivariate Cox regression analysis showed that C3 levels were an independent predictor of mortality. Complement played a pathogenic role in HBV-ACLF patients and C3 was an independent predictor of mortality.

## 1. Introduction

Chronic hepatitis B (CHB) resulting from a variety of hepatic disease processes caused by HBV infection can lead to acute-on-chronic liver failure (HBV-ACLF), which is a severe clinical syndrome characterized by an acute deterioration of liver function with the eventual development of multiple organ failure [1, 2]. A poor understanding of the pathogenesis of HBV-ACLF and lack of effective treatment options result in extremely high mortality rates [1, 2]. There is a growing appreciation that immunity-mediated inflammation plays an important role in the pathogenesis of HBV-ACLF [3]. In particular, different arms of the innate and adaptive immune system make critical contributions to the progression of HBV-ACLF [1]. However, it is not clear if the complement, which is an important bridge between the innate and adaptive immune systems, plays a role in the pathogenesis of HBV-ACLF.

The complement system comprises approximately 30 proteins that are present either as soluble factors or as membrane-associated proteins [4]. The complement can be activated via the classical, lectin, or alternative pathways, resulting in C3 activation and leading to the generation of the membrane attack complex (C5b-9). Complement activation has also been reported to activate multiple immune cells and play an important role in host defense and wound healing by increasing secretion of inflammatory cytokines. However, overactivation of complement components can lead to tissue necrosis and multiorgan dysfunction [5–7].

The role of the complement has been implicated in liver regeneration after partial hepatectomy [8], liver fibrosis [9], ischemia reperfusion [10], alcoholic liver disease [11], nonalcoholic steatohepatitis [12], and viral persistence in patients with CHB or chronic hepatitis C infections [4, 13]. However, there are few studies which investigated its role in patients with HBV-ACLF. An animal liver failure model recently showed activation of complement as evidenced by the hepatic deposition of C3 and C5b-9. Compared with wild-type mice, C3−/− mice survived significantly longer and displayed reduced liver inflammation [14]. Another study using liver specimens from two patients with HBV-ACLF revealed a significant deposition of complement components in the liver parenchyma [15]. Given the observation that complement may be activated in patients with HBV-ACLF, we sought to determine the role of complement in a large sample cohort of Chinese patients with HBV-ACLF and to explore its relationship with disease activity. We tested the hypothesis that complement may play a role in the pathogenesis of HBV-ACLF and could be an independent risk factor for mortality in HBV-ACLF patients.

## 2. Patients and Methods

### 2.1. Study Design and Patients

In this prospective, observational study, we enrolled HBV-ACLF patients who were admitted to our department between April 2009 and March 2010. Adult HBV-ACLF patients who were willing to participate in and consented to the study were screened for this study based on previously described criteria [16]. Among exclusion criteria were (1) presence of other liver diseases including autoimmune liver diseases, Wilson's disease, or evidence of cancer and liver cirrhosis; (2) coinfection with hepatitis A, C, D, or E or HIV virus; (3) treatment with artificial liver support or immunomodulatory drugs within 6 months prior to the screening or during the hospital stay; (4) history of drug abuse or alcohol abuse; and (5) history of renal, cardiovascular, pulmonary, endocrine, or rheumatic diseases. Pregnant women were excluded. Cirrhosis was diagnosed when a small, nodular liver was seen on ultrasound, computerized tomography scans, or magnetic resonance, with the exclusion of primary biliary cirrhosis and cirrhosis caused by schistosome [17]. Clinical assessment and blood samplings were performed at admission and prior to initiation of treatment. Each patient was treated with the same comprehensive supportive treatment (i.e., reduced glutathione, glycyrrhizin, ademetionine, polyene phosphatidylcholine, alprostadil, plasma or albumin transfusion if needed, and antiviral therapy using nucleos(t)ide analogues if HBV-DNA was detected.) [17]. The endpoint of this study was mortality when patients were discharged from our department. We also enrolled 35 CHB patients and 16 healthy controls (NC) from our hospital. CHB were diagnosed according to previously described criteria [16]. Peripheral blood was collected and the serum was separated and analyzed immediately. The study protocol was evaluated and approved by the ethics committee of our hospital, and written informed consent was obtained from each subject prior to the evaluation.

### 2.2. Analysis of C3, C4, and CH50

Serum concentrations of C3 and C4 (the third and fourth components of the complement) were analyzed using kits from Roche Diagnostics (Indianapolis, IN, USA) according to the manufacturer's guidelines. Complement system function (CH50) was evaluated using the liposome immunoassay (Wako Diagnostics, Richmond, VA, USA) according to the manufacturer's guidelines. All values were compared to the normal ranges which were reported as 0.8–1.6 g/L for C3, 0.1–0.4 g/L for C4, and 23–46 U/mL for CH50.

### 2.3. Parameters for Disease Severity

The MELD-Na score was used to assess disease severity and calculated as previously described [18]. If the sodium values were below 125 mmol/L, they were set to 125 mmol/L, and if the values were above 140 mmol/L, they were adjusted to 140 mmol/L. Aspartate aminotransferase (AST), alanine aminotransferase (ALT), total bilirubin (Tbil), albumin (ALB), globulin (GLB), and creatinine (Cr) were quantitated using an autoanalyzer (TBA-30FR Toshiba, Tokyo, Japan). Prothrombin time (PT) and prothrombin time activity (PTA) were measured using an automatic hemostasis/thrombosis analyzer (STA compact, Holliston, MA, USA). Alpha-fetoprotein (AFP) was determined using Roche's Elecsys system (Hoffmann-La Roche, Basel, Switzerland).

### 2.4. Virological Assessment

HBV-DNA levels were quantitated by real-time quantitative PCR using the ABI7300 thermocycler (AppliedBiosystems, Foster City, CA, USA). The limit of detection of the assay was 500 copies/mL. Serum HBV markers, including hepatitis B s antigen (HBsAg), hepatitis B s antibody (HBsAb), hepatitis B e antigen (HBeAg), hepatitis B e antibody (HBeAb), and hepatitis B c antibody (HBcAb), were determined using the Elecsys system (Hoffmann-La Roche, Basel, Switzerland).

### 2.5. Statistical Analysis

All data were analyzed using SPSS version 13.0 software (Chicago, IL, USA) and expressed as frequencies, median, and range or as mean ± standard error. Differences in variables were analyzed using ANOVA and Student's *t*-tests (for normally distributed data) or Kruskal-Wallis and Mann-Whitney *U* tests (for nonnormally distributed data). Comparisons between C3 grades were performed using the Chi-square test or Kruskal-Wallis test when appropriate for frequencies. Categorical data were analyzed using the Chi-square test and Fisher's exact test. Correlation analysis was evaluated by the Spearman rank correlation test. Survival was analyzed by Kaplan-Meier curve. Multivariate analysis was conducted using backward stepwise Cox proportional hazards regression analysis for the probability of mortality. A two-sided *P* < 0.05 was considered statistically significant.

## 3. Results

### 3.1. Characteristics of Patients

**Figure 1:**
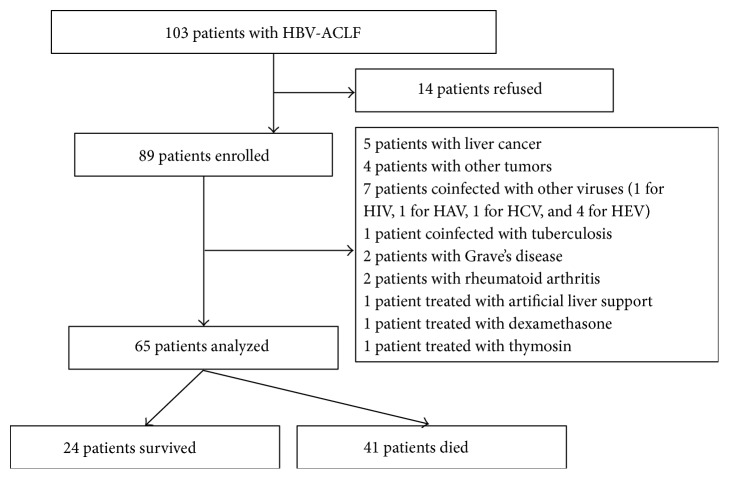
Selection and deposition of patients.

Of a total of 103 consecutive HBV-ACLF patients who met the diagnostic criteria, 14 patients refused to participate in the study and 24 patients were excluded from the final analysis for the following reasons: 9 patients had tumors (5 patients had liver cancer and 4 had other tumors); 7 patients had coinfection with other viruses; 1 patient was coinfected with tuberculosis; 4 patients had systematic autoimmune diseases (2 patients had Grave's disease and 2 had rheumatoid arthritis) and were suspected to have a flare of autoimmune hepatitis; and 3 patients received other medical care (1 received artificial liver support, 1 received dexamethasone, and 1 received thymosin) during their hospital stay. Finally, 65 patients were assigned to the HBV-ACLF group with a MELD-Na score at admission of 31.70 ± 0.48. Among them, 55 patients received antiviral therapy due to the presence of HBV-DNA. Our study patients had a median hospital stay of 39 days (range 3–144). Twenty four patients survived and were discharged from the hospital while 41 patients died. A summary of patients' deposition is shown in Figure 1. The characteristics of patients from the HBV-ACLF, CHB, and NC groups are shown in Table 1. No significant differences existed among three groups for the age and gender ratio (*P* = 0.165 and *P* = 0.531, resp.). Moreover, there were no significant differences in HBeAg levels between the CHB and HBV-ACLF groups (*P* = 0.529).

### 3.2. C3, C4, and CH50 Levels Were Significantly Lower in HBV-ACLF Patients Independently of HBeAg Presence

**Figure 2:**
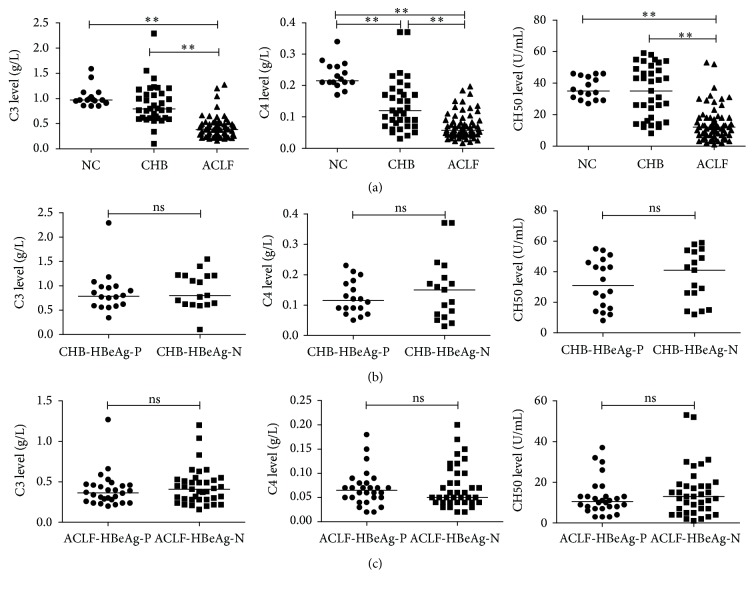
C3, C4, and CH50 levels were significantly lower in HBV-ACLF patients, independently of HBeAg presence. Pooled data indicated the levels of C3, C4, and CH50 in each group, where the lines indicated the median. (a) Serum C3, C4, and CH50 levels were significantly lower in HBV-ACLF patients compared to CHB subjects and NC group. No significant differences existed between patients with HBeAg positive and those with HBeAg negative, neither in CHB group (b) nor in HBV-ACLF group (c). ^*∗∗*^ *P* < 0.01; ns, not significant. ACLF, acute-on-chronic liver failure; CHB, chronic hepatitis B; NC, normal control; HBeAg-P, HBeAg-positive; HBeAg-N, HBeAg-negative.

Complement activation is a complicated process and can be triggered by different pathways. Activation leads to convergence of all complement pathways followed by depletion of the central component C3 [4]. Therefore, we first determined the serum C3 levels in HBV-ACLF patients and controls. HBV-ACLF patients had significantly lower serum C3 levels (median 0.384 g/L) compared to CHB subjects (median 0.795 g/L, *P* < 0.001) and normal controls (median 0.970 g/L, *P* < 0.001; Figure 2(a)). Serum C4 and CH50 levels were also significantly lower in HBV-ACLF patients compared to CHB patients and normal controls (median 0.057 g/L versus 0.116 g/L versus 0.215 g/L; median 12 U/mL versus 35 U/mL versus 35 U/mL, resp., Figure 2(a)). We then determined the correlation between HBeAg presence and complement expression. In the CHB group, no significant differences existed in C3, C4, or CH50 levels between HBeAg-positive (*n* = 18) and HBeAg-negative patients (*n* = 17) (*P* = 0.373, *P* = 0.666, and *P* = 0.338, resp., Figure 2(b)). There was also no significant difference in C3, C4, or CH50 levels between HBeAg-positive HBV-ACLF patients (*n* = 28) and those who were HBeAg-negative (*n* = 37) (*P* = 0.420, *P* = 0.740, and *P* = 0.474, resp., Figure 2(c)).

### 3.3. Lower Complement Levels Indicated Liver Injury and Impaired Liver Regeneration in HBV-ACLF Patients

**Figure 3:**
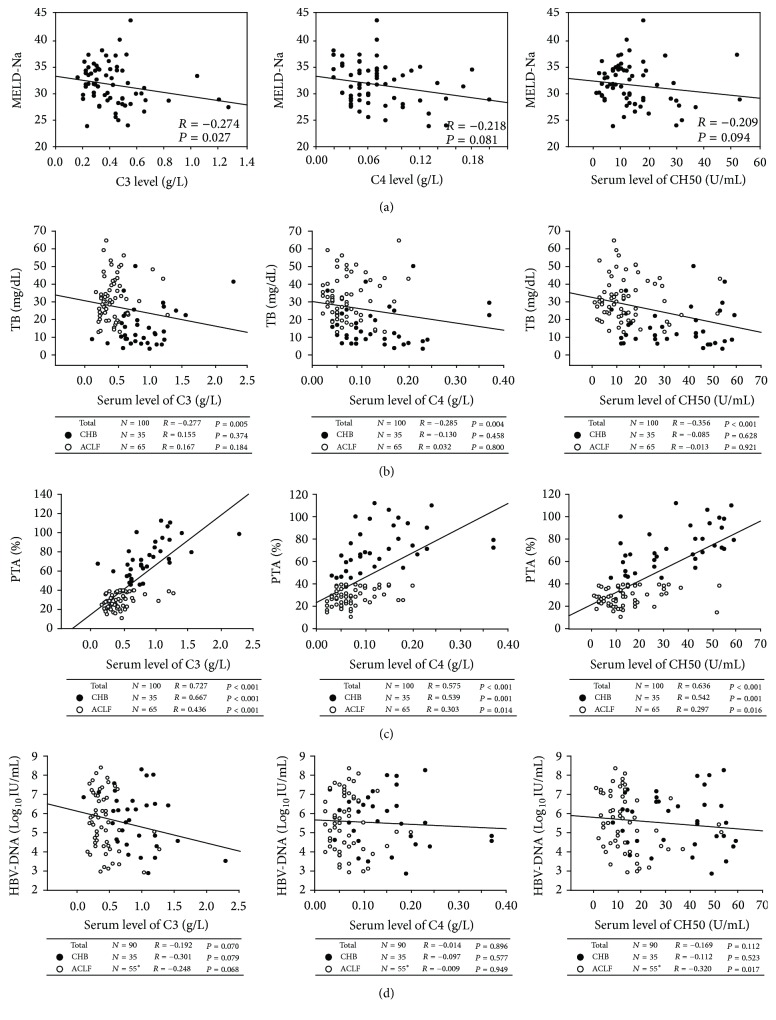
Lower complement levels indicated liver injury in HBV-ACLF patients. A significant negative correlation was found between C3 levels and MELD-Na scores (a). C3, C4, and CH50 levels in serum were negatively correlated with serum Tbil levels (b) but positively associated with plasma PTA levels (c) in HBV-infected patients (chronic hepatitis B (CHB) and acute-on-chronic liver failure (ACLF)). There was a significant negative correlation between CH50 levels and HBV-DNA loads and a slight negative correlation between C3 levels and HBV-DNA loads in HBV-ACLF patients (d). Solid line, linear growth trend; *R*, correlation coefficient. *P* values were shown.

**Figure 4:**
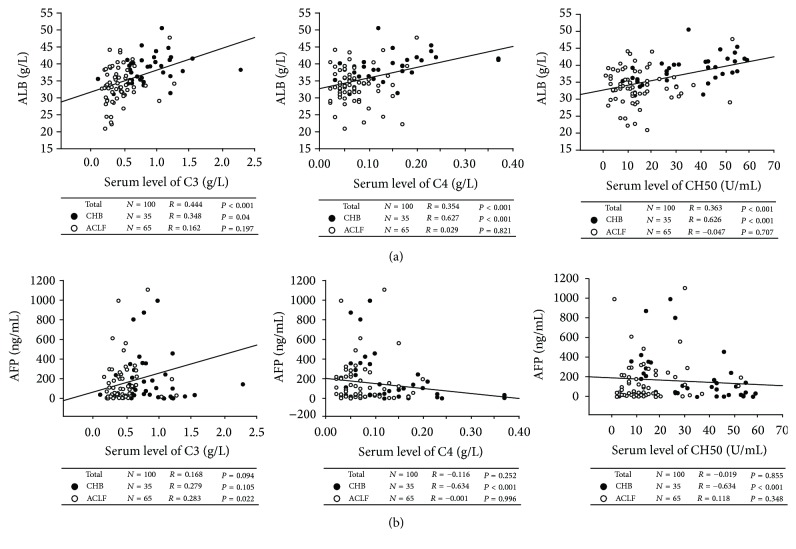
Lower complement levels indicated impaired liver regeneration in HBV-ACLF patients. (a) C3, C4, and CH50 levels in serum were positively associated with serum ALB levels in HBV-infected patients (chronic hepatitis B (CHB) and acute-on-chronic liver failure (ACLF)). (b) A positive correlation was found between serum C3 levels and AFP levels in HBV-ACLF patients. Solid line, linear growth trend; *R*, correlation coefficient. *P* values were shown.

We analyzed the correlation between serum C3, C4, and CH50 levels and parameters for disease severity in HBV-ACLF patients. We showed a significant negative correlation between C3 levels and MELD-Na scores (*r* = −0.274, *P* = 0.027). However, MELD-Na scores were not significantly associated with C4 levels (*r* = −0.218, *P* = 0.081) or CH50 levels (*r* = −0.209, *P* = 0.094; Figure 3(a)). Interestingly, serum Tbil levels were negatively correlated with C3, C4, and CH50 levels (*r* = −0.277, *P* = 0.005; *r* = −0.285, *P* = 0.004; and *r* = −0.356, *P* < 0.001, resp., Figure 3(b)), while plasma PTA levels were positively correlated with C3, C4, or CH50 levels (*r* = 0.727, *P* < 0.001; *r* = 0.575, *P* < 0.001; and *r* = 0.636, *P* < 0.001, resp., Figure 3(c)). There was a significant negative correlation between CH50 levels and HBV-DNA loads (*r* = −0.320, *P* = 0.017) and a slight negative correlation between C3 levels and HBV-DNA loads (*r* = −0.248, *P* = 0.068; Figure 3(d)) in HBV-ACLF patients. We also showed a positive association between serum ALB levels and C3, C4, and CH50 levels (*r* = 0.444, *P* < 0.001; *r* = 0.354, *P* < 0.001; and *r* = 0.363, *P* < 0.001, resp., Figure 4(a)) in these HBV-infected subjects. Furthermore, there was a positive correlation between serum C3 levels and AFP levels (*r* = 0.283, *P* = 0.022; Figure 4(b)) in HBV-ACLF patients. However, no significant correlations existed between C3, C4, or CH50 levels and ALT levels, AST levels, or GLB levels in these HBV-infected patients (data not shown). These findings suggest that lower complement levels were associated with liver injury and impaired liver regeneration in HBV-ACLF patients.

### 3.4. Survival Rate Varied Depending on C3 Levels

**Figure 5:**
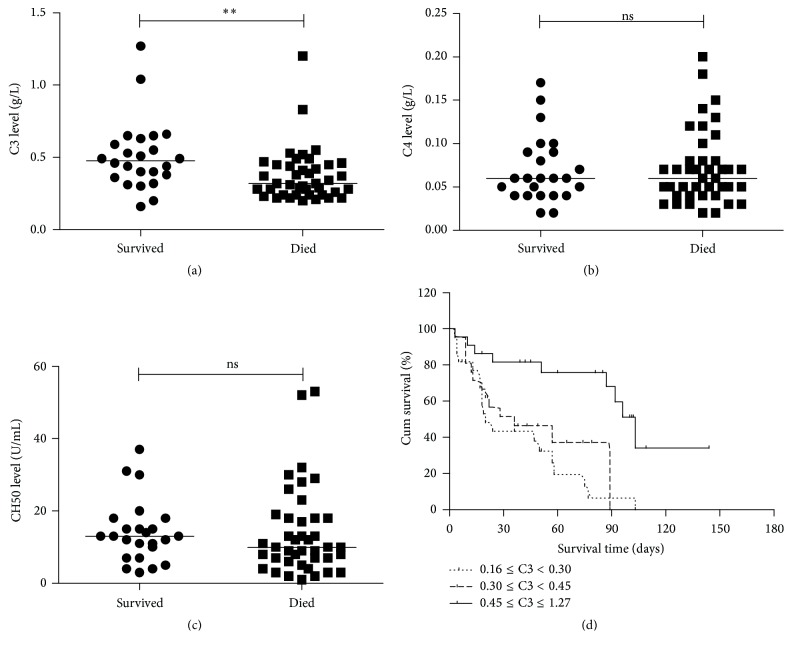
Survival rate varied depending on C3 levels. Pooled data indicated the levels of C3, C4, and CH50 in patients who died (*n* = 41) and those who survived (*n* = 25), where the lines indicated the median. Serum C3 levels were significantly lower in HBV-ACLF patients who died compared to patients who survived (a). However, no significant differences in serum C4 or CH50 levels were found between them (b and c). (d) Using the Kaplan-Meier analysis, the log-rank test revealed that a significant difference existed among three groups according to C3 levels, and patients with a high grade of C3 had a significantly higher survival rate compared to those with moderate grade or low grade (log-rank 15.443, *P* < 0.001). ^*∗∗*^ *P* < 0.01; ns, not significant.

We examined the correlation between clinical outcome and expression of C3, C4, and CH50. Only C3 levels were significantly lower in HBV-ACLF patients who died (median 0.319 g/L, *n* = 41) compared to patients who survived (median 0.472 g/L, *n* = 25, *P* = 0.003; Figure 5(a)). There was no significant difference in C4 or CH50 levels between patients who died and those who survived (*P* = 0.901 and *P* = 0.335, resp., Figures 5(b) and 5(c)). Based on further analysis, we grouped the 65 patients into 3 groups based on C3 values (low grade: 0.16–0.30 g/L, *n* = 22; moderate grade: 0.3–0.45 g/L, *n* = 21; and high grade: 0.45–1.27 g/L, *n* = 22). The minimum C3 was 0.16 g/L, 33th percentile was 0.30 g/L, 66th percentile was 0.45 g/L, and maximum was 1.27 g/L. In a median hospital stay of 39 days (range 3–144), 41 patients (63%) died during the hospital stay of which 19 patients (86%) had low grade C3, 13 patients had (62%) moderate grade C3, and 9 patients (41%) had high grade C3, making a significant difference (*P* = 0.008). A majority of deaths occurred due to the development of multiorgan failure caused by progressive liver failure, leading to hepatorenal syndrome and hepatic encephalopathy. In addition, using the Kaplan-Meier analysis, the log-rank test revealed a significant difference in C3 levels among three groups. Patients with a high grade of C3 had a significantly higher survival rate compared to those with moderate grade or low grade (log-rank 15.443, *P* < 0.001, Figure 5(d)).

### 3.5. C3 Was an Independent Predictor of Mortality

We analyzed the baseline clinical and laboratory variables as possible predictors of mortality. Using univariate Cox regression analysis, we showed that age, MELD-Na score, PTA, AST, Cr, and C3 grade were significantly associated with mortality (Table 2). Using these significant variables in multivariate Cox regression analysis, we showed that MELD-Na score (HR 1.189, 95% CI 1.071–1.320, *P* = 0.001), Cr (HR 1.013, 95% CI 1.001–1.025, *P* = 0.032), and C3 grade (low grade: HR 2.771, 95% CI 1.191–6.446, *P* = 0.018 and moderate grade: HR 3.071, 95% CI 1.201–7.852, *P* = 0.019) were independent baseline predictors of mortality in HBV-ACLF patients.

## 4. Discussion

Although the role of complement has been explored extensively in several liver diseases, surprisingly few reports have examined its role in HBV-ACLF patients. To our knowledge, ours is the first study which extensively explores the role of complement in a large sample cohort of HBV-ACLF patients. We present evidence that (1) C3, C4, and CH50 levels were significantly lower in HBV-ACLF patients, (2) lower complement levels were closely correlated with liver injury and impaired liver regeneration, and (3) mortality varied depending on C3 levels and C3 levels may be an independent predictor of mortality. Collectively, our data support the idea that complement plays a key role in the pathogenesis of HBV-ACLF and C3 may be a potential target for developing pharmaceuticals that interrupt or dampen complement mediated liver injury.

Accumulating data have indicated a pathogenic role of complement in a variety of liver diseases [8–13]. However, the role of the complement in HBV-ACLF patients is poorly understood. Immunohistochemical analysis has shown the deposition of the membrane attack complex around necrotic areas in patients with fulminant hepatitis, which indicates activation of the complement system and contributes to liver injury [19]. In a mouse model of fulminant hepatic failure, C3−/− mice survived longer and displayed reduced liver inflammation and attenuated pathological damage compared with wild-type mice [14]. The complement was also found to be deposited in the liver parenchyma of two HBV-ACLF patients [15]. These studies led us to hypothesize that complement may mediate hepatic injury in liver failure. Our data suggest that complement system was closely correlated with liver injury in HBV-ACLF patients. There are several lines of evidence to support this notion. Firstly, we demonstrated that HBV-ACLF patients had lower serum C3, C4, and CH50 levels compared to CHB patients or healthy individuals. Secondly, C3 levels were negatively correlated with MELD-Na score, which was an important predictor of clinical outcome in HBV-ACLF [18]. Thirdly, serum C3, C4, and CH50 levels in HBV-infected patients were negatively associated with serum Tbil levels but positively correlated with plasma PTA levels, which often serve as markers of liver injury [20]. These results suggest that complement was closely associated with HBV-infection induced liver damage, which may contribute to the pathogenesis of HBV-ACLF.

In addition to its pathogenic role in hepatic injury, recent evidence indicated that complement was required for liver regeneration [21–23]. Using a mouse model of 70% partial hepatectomy, mice deficient in C3 and C5 exhibited impaired liver regeneration and high mortality after liver resection. However, reconstitution of the complement-deficient mice with C3a or C5a improved the regenerative response [22]. Complement activation products C3a and C5a were also shown to play a key role in the proliferative response and hepatocyte regeneration via the TNF-*α* and IL-6 pathways [22]. A similar role for complement and for C3a receptor (C3aR) and C5aR signaling in liver regeneration has been demonstrated in a mouse model of CCL4-induced liver toxicity [21, 23]. Our data were consistent with these studies and showed that complement was necessary for liver regeneration. We showed a positive correlation between C3, C4, and CH50 levels and serum ALB levels and also a positive correlation between serum C3 levels and serum AFP, which is considered to be a valuable marker of hepatocyte regeneration. Taken together, these results suggest that complement plays a crucial role in impairment of liver regeneration. It is important to further study the role of complement in liver regeneration in patients with HBV-ACLF.

A number of studies have suggested that reactivation of HBV infection may be an initial event in inducing the pathogenesis of HBV-ACLF subjects [24–26]. Reactivation is often spontaneous but can also be triggered by cancer chemotherapy, immune suppression, or alteration in immune function. A previous study showed that spontaneous acute exacerbation of CHB infection was seen with a cumulative probability of 15%–37% after 4 years of follow-up [27]. As a result, APASL guidelines strongly recommend initiation of antiviral therapy in HBV-ACLF patients [28]. A recent meta-analysis showing that nucleoside analogues can significantly improve the survival of HBV-ACLF patients [29] highlighted the role of HBV replication in HBV-ACLF. In our study, CH50 levels and C3 levels were negatively correlated with HBV-DNA loads at admission prior to receiving antiviral therapy. The data indicated that HBV replication, at least in part, may participate in the complement mediated response and may be a contributor to liver injury. However, it will be important to further study the direct interaction between HBV replication and complement mediated inflammation.

Although antiviral therapy using nucleoside analogues is an important aspect of treatment aimed at inhibiting HBV-DNA replication, the outcome of HBV-ACLF patients is still extremely poor. In our study, 55 HBV-ACLF patients with detectable HBV-DNA were treated with antiviral therapy. However, the mortality in this cohort was as high as 63% which was consistent with data from another report [1]. These data emphasize the limitations of current comprehensive care and underscore the importance of developing novel treatment strategies. Previous studies using different animal models showed that C3 plays a pathogenic role in various liver diseases. C3 deficiency or C3a antagonists improved survival in alcoholic liver disease or in liver failure models [11, 14]. Interestingly, we showed that mortality was significantly lower in patients with a high grade of C3 compared to patients with moderate grade, while patients with a low C3 grade had the highest mortality. In addition, we showed that C3 levels at admission were an independent risk factor of mortality. Taken together, these data emphasize the importance of C3 in the pathogenesis of HBV-ACLF and suggest that it could be a target for immunotherapy intervention.

This study has two important limitations. Firstly, we did not analyze the complement levels in the microenvironment of liver tissues in HBV-ACLF patients due to poor coagulation, which may be a contraindication for liver biopsy. It is important to further investigate the relationship between the microenvironment of liver tissues and the circulating complement levels. Secondly, this is a descriptive and observational study showing that complement system was involved in the pathogenesis of HBV-ACLF and C3 levels at admission were a predictor of mortality in HBV-ACLF patients. Future studies to explore the mechanisms underlying the role of complement in HBV-ACLF are urgently needed.

In summary, we showed that (1) complement components were significantly decreased in HBV-ACLF patients, suggesting that the complement may be implicated in the pathogenesis of HBV-ACLF; (2) lower complement was closely correlated with liver injury and impaired liver regeneration; and (3) the survival rate varied depending on C3 levels and C3 levels at admission were an independent risk factor of mortality in HBV-ACLF patients. Our findings shed new light on the pathogenic mechanisms involved in pathogenesis of HBV-ACLF and an approach of modulated C3 represents a potential therapeutic strategy for patients with HBV-ACLF.

## Figures and Tables

**Table 1:** Clinical characteristics of the populations enrolled in the study.

Variables	NC (*n* = 16)	CHB (*n* = 35)	HBV-ACLF (*n* = 65)
Sex (male)	14	32	62
Age (years)	37.69 ± 2.93	37.69 ± 1.28	41.52 ± 1.47
ALT (U/L)	23.68 ± 2.30	529 (41–2424)	257 (25–1986)
AST (U/L)	23.56 ± 1.84	386 (55–2646)	225 (78–3023)
Tbil (mg/dL)	N.D.	9.50 (0.92–49.71)	28.82 (10.75–64.82)
PT (s)	N.D.	15.6 (12.5–21.2)	28.0 (20.2–60.6)
PTA (%)	N.D.	72 (46–113)	29 (11–40)
ALB (g/L)	N.D.	38.70 ± 0.69	33.88 ± 0.66
GLB (g/L)	N.D.	29.56 ± 0.87	31.58 ± 0.90
Na (mmol/L)	N.D.	139.0 (133.8–142.5)	137.2 (117.7–146.2)
Creatinine (*μ*mol/L)	N.D.	64.0 (42–117)	63.4 (37.5–267)
AFP (ng/mL)	N.D.	108.0 (1.7–1000)	53.5 (2.8–1112)
DNA (log_10_IU/mL)	N.D.	5.69 ± 0.24	5.58 ± 0.20^*∗*^
HBsAg-positive	0	35	65
HBsAb-positive	16	0	0
HBeAg-positive	0	18	28
HBeAb-positive	0	17	37
HBcAb-positive	0	35	65

Data are shown as means and standard error or median and range. ACLF, acute-on-chronic liver failure; CHB, chronic hepatitis B; NC, normal control; ALT, alanine aminotransferase; AST, aspartate aminotransferase; PT, prothrombin time; ALB, albumin; GLB, globulin; AFP, alpha-fetoprotein; and N.D., not determined. ^*∗*^HBV-DNA could not be detected in 10 patients with HBV-ACLF.

**Table 2:** Factors associated with mortality in univariate Cox regression analysis.

Variables	*P* value	Hazard ratio	95% CI
Age (years)	0.032	1.029	1.002–1.057
HBeAg-positive	0.190	1.538	0.807–2.931
MELD-Na	<0.001	1.206	1.100–1.322
Tbil (mg/dL)	0.490	1.009	0.984–1.034
PTA (%)	<0.001	0.919	0.880–0.959
AST (U/L)	0.019	1.001	1.000–1.001
ALT (U/L)	0.242	1.000	1.000–1.001
ALB (g/L)	0.631	1.014	0.958–1.074
GLB (g/L)	0.912	1.002	0.964–1.042
Creatinine (*μ*mol/L)	0.002	1.017	1.006–1.028
AFP (ng/mL)	0.753	1.000	0.998–1.001
DNA (log_10_IU/mL)	0.061	1.004	1.000–1.009
C3 (g/L)	0.001		
0.16 ≤ C3 < 0.3	<0.001	4.625	2.018–10.600
0.3 ≤ C3 < 0.45	0.007	3.483	1.402–8.653
C4 (g/L)	0.816	2.566	0.001–7060.782
CH50 (U/mL)	0.742	0.994	0.960–1.030
Antiviral therapy	0.085	1.811	0.921–3.557

AST, aspartate aminotransferase; ALT, alanine aminotransferase; ALB, albumin; GLB, globulin; and AFP, alpha-fetoprotein.

## Abbreviations

ACLF: Acute-on-chronic liver failure
CHB: Chronic hepatitis B
NC: Normal control
HBV: Hepatitis B virus
C3: The third complement component
C4: The fourth complement component
PTA: Prothrombin time activity
Tbil: Total bilirubin.
